# A Comparison of Patients from Argentina and Germany to Assess Factors Impacting Brachial Plexus and Brain Injury

**DOI:** 10.1055/s-0039-1693687

**Published:** 2019-08-13

**Authors:** Mariano Socolovsky, Gregor Antoniadis, Ana Lovaglio, Gregor Durner, Gonzalo Bonilla, Markus Schmidhammer, Gilda di Masi

**Affiliations:** 1 Peripheral Nerve and Plexus Program, Department of Neurosurgery, University of Buenos Aires School of Medicine, Buenos Aires Argentina; 2 Peripheral Nerve Surgery Unit, Department of Neurosurgery, University of Ulm, Ulm, Germany

**Keywords:** brachial plexus injuries, epidemiological comparison, motorcycle accident, demography

## Abstract

**Background** Traumatic brachial plexus injuries (BPIs) represent a major cause of disability in young patients. The purpose of this study was to compare two populations (from Argentina and Germany) who suffered a traumatic BPI after a motorcycle accident to identify predictors of BPI and brain injury severity.

**Methods** Univariate and multivariable intergroup comparisons were conducted, and odds ratios were calculated to assess the associations between the different demographic, morphometric, and trauma-related variables, and the type and severity of patients' injuries. Pearson correlation coefficients were generated to identify statistically significant correlations.

**Results** A total of 187 patients were analyzed, 139 from Argentina and 48 from Germany. The two countries differed significantly in age and several morphometric and trauma-related variables. The clinical presentation was also convincingly different in the two countries. The following three variables remained as statistically significant predictors of a complete (vs. partial) BPI: living in Argentina ( *p* < 0.001), presenting prior to 2015 ( *p* = 0.004), and greater estimated speed at the time of impact ( *p* = 0.074). As for BPIs, a disproportionate percentage (85.6%) of more severe brain injuries occurred in Argentinian patients ( *p* < 0.001) and among those whose accident involved striking a stationary vertical object.

**Conclusions** This study identified several factors that might be considered when planning governmental policies and education initiatives to reduce BPI and brain injuries related to motorcycle use.

**Level of evidence** II-2 (evidence obtained from case–control studies).

## Introduction

Traumatic brachial plexus injuries (BPIs) are a tremendous economic burden, as they affect mostly young people during their early working years. 1 2 3 4 They are primarily, but not exclusively, caused by motorcycle accidents. Recently, reports have been published on how patients' body mass index (BMI), 5 6 7 and mechanism of trauma 8 9 10 impact the results of reconstructive brachial plexus surgery.

For this study, we decided to analyze two patient populations, an ocean apart, who suffered from traumatic BPI after a motorcycle accident: one from Argentina and the other from Germany. We theorized that factors such as a patient's size and gender, the engine size of the motorcycle and its speed at the moment of impact, whether the rider was wearing a helmet or not, and the underlying mechanism of trauma (i.e., whether the patient struck the ground, an oncoming vehicle, or a stationary vertical object during the accident) could ultimately impact the severity of BPI and brain injuries differentially in the two different countries. Specifically, we sought to evaluate the role of each of the aforementioned variables in determining if a patient's BPI was partial or complete and if the brain injury was nonexistent-to-mild or moderate-to-severe.

Specific objectives were to compare patients from Argentina and Germany with respect to the impact upon injury: (1) year of injury, (2) demographic and morphometric characteristics, (3) helmet use, (4) mechanism of trauma (e.g., hitting the ground vs. a vertical pole), and (5) motorcycle engine size. We also sought to assess (6) the association between brain and plexus injury severity, (7) the association between helmet use and BPI severity, specifically in patients whose accident involved striking the ground, and, finally, to identify (8) any other clinically important and statistically significant correlations between the various demographic, morphometric, and injury-related variables. As the two countries might differ in many epidemiological and demographical variables, we believed that performing this type of analysis was worthwhile, as it could provide useful information that could aid in the planning of governmental policies and education initiatives to reduce the societal impact of motorcycle accidents.

## Methods

### Inclusion and Exclusion Criteria

This study was orchestrated at two separate neurosurgery units, one in Argentina and the other in Germany. To create this comparison, we established an observation period of 12 months (October 2016 to September 2017). Over that period, all patients who presented to either one of the neurosurgery units, whether presenting preoperatively or returning for follow-up postmotorcycle accident-related BPI, were included in this study. Note that some of these patients had had their injury years previously and were merely being assessed during follow-up. Such patients had their charts reviewed and were asked a series of specific questions (either verbally or in written form) to collect data on their gender, age, and weight at the moment of the accident, height, the clinical description of the palsy (e.g., C56, C567, C8T1), the presence of traumatic brain injury and its classification, the object struck (whether the ground, a vertical pole, another vehicle, or other), whether they were using a helmet or not at the moment of impact, the estimated speed of the motorcycle at that moment, and the engine size of the motorcycle. The study excluded were patients who (1) lacked any one of the aforementioned data variables (except estimated speed at the moment of impact), (2) did not have their BPI confirmed, whether by imaging (computed tomography [CT] or magnetic resonance imaging [MRI]) preoperatively, inspection intraoperatively, or both, or (3) refused to participate in this study, for which informed consent was necessary.

### Data Analysis Methods

All characteristics of the subject sample are presented as either means with standard deviations for continuous variables or as absolute numbers and percentage of the sample for categorical variables. Univariate intergroup comparisons were conducted—comparing patients with versus without helmets, patients with complete versus partial BPIs, and patients with mild-to-no brain injury versus patients with moderate to severe brain injury—using Student's tests for continuous variables and Pearson's *χ* ^2^ analysis for categorical (nominal or ordinal) variables, with Levene's test for homogeneity of variances performed for all continuous variables ad hoc, and the conservative Welch-Satterthwaite method performed to adjust degrees of freedom (df) for noncontinuous variables. For all univariate comparisons, *p* ≤ 0.05 was set as the criterion, indicating a statistically significant difference, and *p* between 0.051 and 0.10 was set as the range for borderline significance. All tests were two-tailed.

To further identify characteristics that distinguish groups from one another, multivariable analysis was conducted by constructing and statistically testing two stepwise (hierarchical) binary logistic regression models, one for each of the two main dependent variables of interest: partial versus complete plexus injury, and mild/absent versus moderate/severe brain injury. Step 1 for each model consisted of forward entry of demographic variables, Step 2 of event-related variables as independent, with all variables with p ≤ 0.10 retained in the final model.

Odds ratios (ORs) were calculated to assess the associations between helmet use and BPI (incomplete/complete), helmet use and brain injury (none/mild vs. moderate/severe), and BPI and brain injury.

To identify correlations between variables, Pearson correlation coefficients were constructed and tested for statistical significance, with *p* ≤ 0.05 set as the criterion indicating a statistically significant correlation, and the following ranges of *r* set as indicators of correlation strength: *r* ≤ 0.30, weak; *r* = 0.31 to 0.70, intermediate; *r* > 0.70, strong.

All analyses were conducted using the Statistical Package for the Social Sciences (SPSS) version 25 (SPSS Inc.).

## Results

A total of 187 patients were available for analysis, 139 from Argentina and 48 from Germany. All but five (all from Germany) had confirmed nerve-root injury as the source of palsy on MRI scan; these five had their injury confirmed intraoperatively. None of our patients sustained any spine injury. Only three patients across the entire sample, all from Argentina, sustained their trauma prior to the year 2010, with increasing numbers thereafter (40.6% between 2010 and 2014 and 57.8% between 2015 and 2017). Demographic and morphometric characteristics of the two samples are summarized in Table 1 . Note that Germans were a mean 9.4 years older, 7.3 cm taller, and 9.3 kg heavier (all *p* < 0.001) than their Argentinian counterparts.

**Table 1:** Demographics and morphometrics of the sample: overall and by country

	Overall	Argentina	Germany	*χ* ^2^ or *t*	df	*p* -Value
*N*	187	139	48			
Year of presentation						
Pre-2010	1.6%	2.2%	0.0%	*χ* ^2^ = 1.45	2	*p* = 0.49
2010–2014	40.6%	41.7%	37.5%			
2015–2017	57.8%	56.1%	62.5%			
Demographics and morphometrics						
Males	94.9%	94.9%	93.5%	*χ* ^2^ = 0.14	1	0.71
Mean age (years)	28.8	26.3	35.7	*t* = 3.69	54(adjusted)	<0.001
Mean height (cm)	174.2	172.3	179.6	*t* = 5.64	185	<0.001
Mean weight (kg)	76.9	74.0	85.3	*t* = 4.43	185	<0.001
Mean BMI	25.3	24.9	26.4	*t * = 1.84	185	0.068
Underweight	4.3%	4.3%	4.2%	*χ* ^2^ = 4.31	3	0.23
Normal weight	45.5%	49.6%	33.3%			
Overweight	38.5%	36.0%	45.8%			
Obese	11.8%	10.1%	16.7%			

Abbreviation: df, degrees of freedom.

The clinical presentation of patients was also convincingly different in the two countries, with Germans averaging fewer injured roots, over 7.5 times less likely to have a complete plexus injury, and less than half as likely to have at least a moderate-to-severe brain injury than Argentinians (all *p* < 0.001). The two countries were quite different in certain aspects of their accident, with all Germans, but only just over half of Argentinians, reporting that they were wearing a helmet at the time of their motorcycle accident ( *p* < 0.001); Germans vastly more likely to drive motorcycles with larger engines ( *p* < 0.001); and Germans more likely to have struck another vehicle versus a stationary vertical object (like a tree, pole or wall) than Argentinians ( *p* = 0.02). Despite their larger-engine vehicles, Germans were not driving faster (by self-report) at the time of their accident, and no greater percentage were driving at a speed of >80 km/hour ( *p* = 0.98).

Given the clear difference in the two primary measures of clinical status (incomplete vs. complete plexus injuries, and mild/no vs. moderate/severe brain injury) in the two countries, with better status favoring the Germans, combined with the universal use of helmets by Germans versus just over half of Argentinians, demographics, morphometrics, clinical presentation, and injury characteristics were compared between those wearing ( *n* = 120) and not wearing ( *n* = 66) helmets ( Table 2 ). More than 60% of Argentinian patients who claimed to wear a helmet were seen from 2015 onward, whereas there was a pretty even 50–50 split (pre-2015 vs. 2015 + ) among Germans ( *p* = 0.049). Those wearing a helmet averaged 6.2 years older ( *p* < 0.001) and 3.8 cm taller ( *p* = 0.002) than their nonhelmeted counterparts. The two groups did not differ in gender, average weight, BMI, or BMI category.

**Table 2:** Demographics and morphometrics in patients wearing versus not wearing a helmet

	Helmet	No helmet	*χ* ^2^ or *t*	df	*p* -Value
*N*	120	66			
Year of presentation					
Pre-2010	0.0%	4.5%	*χ* ^2^ = 6.01	2	0.049
2010–2014	39.2%	42.4%			
2015–2017	60.8%	53.0%			
Demographics and morphometrics					
Argentina	60.0%	100.0%	*χ* ^2^ = 35.58	1	<0.001
Germany	40.0%	0.0%			
Males	96.6%	90.9%	*χ* ^2^ = 2.63	1	0.11
Mean age (years)	31.0	24.8	*t* = 4.21	181(a)	<0.001
Mean height (cm)	175.5	171.7	*t* = 3.09	184	0.002
Mean weight (kg)	77.9	75.2	*t* = 1.08	184	0.28
Mean BMI	25.3	25.5	*t * = 0.33	184	0.75
Underweight	5.8%	1.5%	*χ* ^2^ = 2.53	3	0.47
Normal weight	42.5%	50.0%			
Overweight	40.0%	36.4%			
Obese	11.7%	12.1%			

Abbreviation: df, degrees of freedom.

In Germany, all patients, male and female, were reported to have been wearing a helmet at the time of their accident. But, in Argentina, while 70 of the 132 males (53%) reported wearing a helmet, only 1 of the 7 females did (14.3%). However, neither complete BPIs (55.1 vs. 70.1%; *χ* ^2^ = 0.85; df = 1; *p* = 0.36) nor moderate/severe brain injuries (48.3 vs. 50.0%; *χ* ^2^ = 0.86 df = 1; *p* = 0.69) differed between the sexes. To seek a potential explanation for this, males and females were compared in regard to estimated speed, nature of object struck (ground, vertical stationary object, another vehicle), and engine size. Women averaged driving an estimated 15 km/hour slower than males, but this difference failed to achieve statistical significance ( *t* = 1.78; df = 155; *p* = 0.076). There also was no gender difference in the object struck ( *χ* ^2^ = 0.89; df = 2; *p* = 0.64) or relative engine size ( *χ* ^2^ = 0.24; d = 2; *p* = 0.89). Just considering riders not wearing a helmet (all in Argentina), again no gender differences were apparent, with 37 of 60 nonhelmeted males (61.7%) versus 4 (66.7%) of 6 nonhelmeted females suffering a complete BPI ( *χ* ^2^ = 0.06, df 1, *p* = 0.81), and 38 of 60 nonhelmeted males (63.3%) and again 4 (66.7%) of 6 nonhelmeted females suffering at least a moderate brain injury ( *χ* ^2^ = 0.03; 1, *p* = 0.87).

Across the entire sample of patients (Argentina and Germany), those wearing a helmet were less likely to have a complete plexus injury (40.8 vs. 62.1%; *p* = 0.005). They also averaged 0.42 fewer injured roots ( *p* = 0.06) than their nonhelmeted counterparts, though this difference just failed to achieve statistical significance. They were more likely to drive a large-engine (≥ 500 cc) motorcycle (27.7 vs. 0%; *p* < 0.001).

More than two-thirds (67.7%) of the patients presenting with a partial BPI did so from 2015 through 2017 versus less than half (47.3%) of those with a complete BPI ( *p* = 0.02) ( Table 3 ). Only 4 (4.4%) of the 91 patients who presented with a complete BPI were from Germany ( *p* < 0.001). Interestingly, those with complete BPI were statistically younger ( *p* = 0.03), shorter ( *p* = 0.01), and lighter ( *p* = 0.005). Those who suffered a complete BPI were also less likely to report wearing a helmet ( *p* = 0.005), less likely to be riding a large-engine motorbike (4.4 vs. 30.8%; *p* < 0.001), and more likely to have a concomitant moderate-to-severe brain injury (70.3 vs. 41.7%; *χ* ^2^ = 15.55; df = 1; *p* < 0.001) ( Table 4 ).

**Table 3:** Demographics and morphometrics in patients with incomplete versus complete PIs

	Partial PI	Complete PI	*χ* ^2^ or *t*	df	*p-* Value
*N*	96	91			
Year of presentation					
Pre-2010	1.0%	2.2%	*χ* ^2^ = 8.02	2	0.018
2010–2014	31.3%	50.5%			
2015–2017	67.7%	47.3%			
Demographics and morphometrics					
Argentina	54.2%	95.6%	*χ* ^2^ = 42.0	1	<0.001
Germany	45.8%	4.4%			
Males	94.4%	94.4%	*χ* ^2^ = 0.30	2	0.30
Mean age (years)	30.5	26.9	*t* = 2.20	156(a)	0.03
Mean height (cm)	175.7	172.6	*t* = 2.57	185	0.011
Mean weight (kg)	80.0	73.6	*t* = 2.83	164(a)	0.005
Mean BMI	25.9	24.7	*t * = 1.74	167(a)	0.085
Underweight	4.2%	4.4%	*χ* ^2^ = 0.71	3	0.19
Normal weight	41.7%	49.5%			
Overweight	37.5%	39.6%			
Obese	16.7%	6.6%			

Abbreviations: df, degrees of freedom; PI, plexus injury.

**Table 4:** Clinical presentation and injury characteristics in patients with an incomplete versus complete PI

	Partial PI	Complete PI	*χ* ^2^ or *t*	df	*p* -Value
*N*	96	91			
Clinical presentation					
No brain injury	32.3%	13.2%	*χ* ^2^ = 16.75	3	0.001
Mild brain injury	26.0%	16.5%			
Moderate brain injury	13.5%	27.5%			
Severe brain injury	28.1%	42.9%			
Injury characteristics					
Wearing a helmet	74.0%	54.4%	*χ* ^2^ = 7.73	1	0.005
Striking the ground	44.7%	41.6%	*χ* ^2^ = 0.83	2	0.66
Striking a stationary vertical object	19.1%	24.7%			
Striking another vehicle	36.2%	33.7%			
Mean motorcycle speed	64.8	70.3	*t* = 1.43	156	0.16
Driving < 40 km/h	8.6%	7.7%	*χ* ^2^ = 0.50	2	0.78
Driving 40–79 km/h	45.7%	41.0%			
Driving ≥ 80 km/h	45.7%	51.3%			
Engine size < 500 cc	69.1%	95.6%	*χ* ^2^ = 21.88	2	<0.001
Engine size 500–999 cc	19.1%	2.2%			
Engine size ≥ 1,000 cc	11.7%	2.2%			
No-to-mild brain injury	58.3%	29.7%	*χ* ^2^ = 15.55	1	<0.001
A moderate-to-severe brain injury	41.7%	70.3%			

Abbreviations: df, degrees of freedom; PI, plexus injury.

**Fig. 1:**
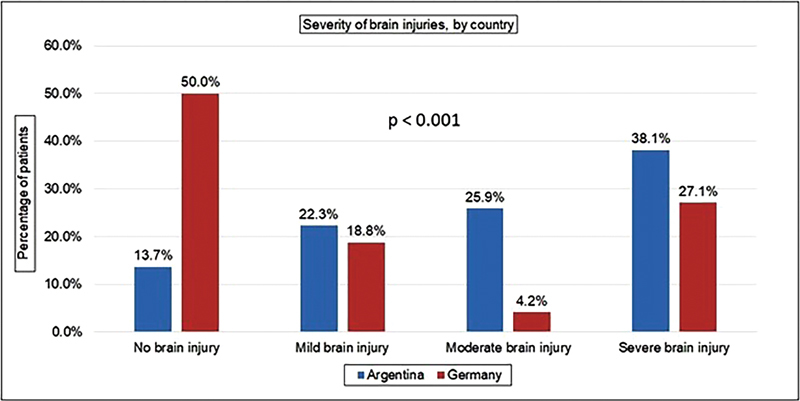
Distribution of brain injuries by severity and country.

On hierarchical binary logistic regression, three variables remained in the model, with *p* ≤ 0.01, as potential predictors of a complete BPI: living in Argentina ( *p* < 0.001), presenting prior to 2015 ( *p* = 0.004), and greater estimated speed at the time of the accident ( *p* = 0.074).This model predicted 27.1% of the variance in the dependent variable, and overall correctly predicted injury severity 72% of the time. Fig. 1 depicts the different distributions of BPI severity between the two countries.

Of the 187 patients, 83 either had no or at most a mild brain injury, whereas the remaining 104 had brain injuries that were rated moderate-to-severe. As with incomplete BPIs, roughly two-thirds (63.9%) of the patients who presented with either no or a mild brain injury did so from 2015 onward. However, contrary to complete BPIs, a majority of the moderate-to-severe brain injuries presented after 2014; therefore, the two patient groups (no/mild vs. moderate/severe brain injury) were not statistically different in the year of presentation ( *p* = 0.19). As for BPIs, a disproportionate percentage (85.6%) of more-severe brain injuries occurred in Argentinian patients ( *p* < 0.001). Otherwise, the two patient groups defined by brain injury severity were not statistically different in any demographic or morphometric measure.

With respect to characteristics of the accident, those with more severe brain injuries were more likely to have struck a stationary vertical object (27.2 vs. 15.0%) and less likely to have struck the ground (35.9 vs. 52.5%) than their milder brain injury counterparts ( *p* = 0.046). On clinical presentation, they averaged more injured roots (4.05 vs. 3.45; *p* = 0.009) and were almost twice as likely to have a concomitant complete plexus injury (61.5 vs. 32.5%; *p* < 0.001). The odds of someone with a moderate-to-severe brain injury also having a complete BPI were greater than three (OR = 3.32), and this was significantly different than 1.0 (95% confidence interval [CI] = 1.81, 6.08).

By hierarchical binary logistic regression, the two statistically significant predictors of a moderate-to-severe brain injury were being injured in Argentina ( *p* = 0.008) and striking a stationary vertical object ( *p* = 0.033).

Table 5 summarizes those 79 patients whose injury involved striking the ground (as opposed to a stationary vertical object or another vehicle). This analysis was performed due to the suspicion that helmets might worsen the risk of BPI among those with this nature of injury. Among these 79, 48 reported having been wearing a helmet, whereas 31 reported that they had not. Among the 31 who reported they had not been wearing a helmet, 18 (58.1%) presented with a complete BPI; among the 48 with a helmet, 19 (39.6%) had a complete BPI. As such, the odds of having a complete BPI was greater not wearing a helmet, though the difference failed to be statistically significant (OR = 2.13; 95% CI = 0.84, 5.26).

**Table 5:** Summarizing riders who struck the ground

Variable	Number	Percentage
*N*	79	
Argentina	63	79.7
Germany	16	20.3
Male	73	94.8
Female	4	5.2
Pre-2010	1	1.3
2010–2014	27	34.2%
2015–2017	51	64.6%
Underweight	2	2.5%
Normal weight	41	51.9%
Overweight	27	34.2%
Obese	9	11.4%
Helmet worn, yes	48	60.8%
Helmet worn, no	31	39.2%
Riding speed < 40 km/h	8	10.1%
Riding speed 40–79 km/h	31	39.2%
Riding speed ≥ 80 km/h	31	39.2%
Engine size < 500 cc	66	83.5%
Engine size 500–999 cc	5	6.3%
Engine size ≥ 1,000 cc	6	7.6%
Partial/no plexus injury	42	53.2%
Complete plexus injury	37	46.8%
Mild/no brain injury	42	53.2%
Moderate/severe brain injury	37	46.8%

## Discussion

### Comparing Argentinian and German Patients

The patient cohorts in these two countries are highly different demographically in terms of age (Germans almost a decade older) but not gender, morphometrically (Germans considerably taller and heavier), in terms of characteristics of their accident (Germans riding more powerful bikes but not faster; all versus only about half of Argentinians wearing helmets; more likely to strike another vehicle), and in terms of clinical presentation (Germans generally having less severe BPIs and brain injuries).

Given the stark difference in helmet use between the two countries, those reporting to have been wearing versus not wearing a helmet were compared across the entire sample. Not surprisingly, given that twice the percentage of Germans as Argentinians wore helmets and that Germans generally were appreciably older, taller, and heavier, those wearing helmets were also older, taller, and heavier than those without helmets.

There are cultural factors behind the lack of use of helmets in Argentinian motorcycle riders. Although wearing helmets is mandated by law in Argentina, as seen in our patient cohort, many fail to observe this law. In our sample, Argentinian brachial plexus patients exhibited more severe injuries, involving both the brain and brachial plexus. At the same time, they generally used smaller vehicles that accelerate well but fail to brake as well. The use of alcohol or other drugs while driving, details that were impossible to collect owing to legal and social reasons, was possibly another factor involved in the increased severity of brachial plexus trauma and the inadequacy of helmet use in Argentina; though the use of helmets is highly enforced in many cities in Argentina, there is marked variability nationwide.

Also analyzed in this paper was the theory that wearing helmets could be linked to more severe BPIs. When the head is covered by a helmet, its diameter is increased. When patients strike the ground, hypothetically the traction inflicted upon plexus structures could be intensified due to this increase in “head + helmet” circumference. Given that Argentinian riders not wearing a helmet generally sustained more severe BPI than those wearing a helmet, our data suggest that this conjecture—that wearing a helmet predisposes someone to more severe BPI—is false. In our dataset, the odds of having a complete BPI was greater when not wearing a helmet, though the difference failed to be statistically significant; the reason behind this finding was potentially linked to the type of trauma rather than the use versus nonuse of protective headgear.

Interestingly, of the just seven female Argentinians in the sample, only one wore a helmet versus 54% of Argentinian males. However, these Argentinian women suffered no worse BPI or brain injuries, possibly because these seven women also reported driving an average of 20 km/hour slower than males. Moreover, on binary logistic regression, wearing a helmet dropped out of a model constructed of demographic, morphometric, and accident variables to predict BPI, whereas country was the strongest predictor. This suggests that other international differences beyond helmet use were more influential: perhaps greater experience riding a motorcycle; heavier bikes actually being protective; and other protective gear more frequently used among Germans, etc. Discouragingly, the use of helmets does not appear to be increasing in Argentina, with still roughly 50% going without.

### Predictors of Brachial Plexus and Brain Injury Severity among Motorcycle Accident Victims in Argentina and Germany

The typical trauma BPI patient is a young male who suffers a motorcycle accident and develops either a complete (more frequent) or incomplete (less frequent) injury. 11 12 In this paper, we have compared data from two different countries. The results of demographic and morphometric comparisons between those with partial and those with complete BPI mimic the differences between Germans and Argentinians, which makes sense. Wearing a helmet was linked to partial BPI as was, surprisingly, having a larger-engine motorcycle. Having a complete BPI was associated with a greater likelihood of a moderate or severe (vs. no or mild) brain injury. On regression analysis, earlier year of injury, country (being in Argentina), and faster motorcycle speed remained predictors of complete BPI, whereas helmet use dropped out, implying that other factors, besides helmet use, might explain the difference between Argentina and Germany.

Other than country, no demographic or morphometric variable was linked to more severe brain injuries on bivariate analysis. Striking the ground was associated with fewer more-severe brain injuries, whereas striking a stationary vertical object such as a wall, pole, or tree was associated with a greater likelihood of more severe brain injuries. Having a moderate-to-severe brain injury was linked to more injured nerve roots and more complete BPI. On regression analysis, living in Argentina and striking a stationary vertical object remained as predictors of more severe brain injury. Again, wearing a helmet dropped out. Why this is so might relate to the absence of mortality data in our samples. It might be, for example, that those not wearing a helmet who survive a motorcycle accident tend to be those whose head did not directly strike the ground, a stationary object, or any other hard surface.

Our study has clear limitations including the combined retrospective and prospective nature of data collection, most data being collected by self-report and not verified objectively, differences in the nature of data collection in the two countries, and the absence of data on other factors (e.g., socioeconomics, education level, mortality) that might have clarified certain associations. A larger sample also might have identified helmet nonuse as a predictor of more severe brain injury, especially since all within the German sample wore helmets.

Nonetheless, we feel that our findings could play a role in the planning of governmental policies and information campaigns aimed toward the prevention of motorcycle-related head and neck injuries. For instance, despite our data's failure to link nonhelmet use with more severe BPI, it has been linked to more severe head injuries; yet current strategies to increase helmet use in Argentina appear to have resulted in little to no change over time. Moreover, given that hitting a stationary object was the type of trauma linked to more severe BPI and brain trauma, objects such as signposts and barriers could and perhaps should be moved further away from the road, especially in Argentina.

## Conclusions

Among those with motorcycle-related BPIs, two countries, Germany and Argentina, appear to be very different in the personal characteristics and practices of their motorcycle riders and the nature of their injuries. Germans are older, taller, and heavier, are twice as likely to wear a helmet (virtually all doing so), have larger-engine vehicles but do not drive faster, and are less likely to suffer a complete BPI or moderate-to-severe brain injury. Interestingly, on multivariable analysis, helmet use did not predict more severe brain injury, implying that other factors such as cultural differences and the nature of the injury may be more important. Further international comparisons could aid in clarifying the role of these and other factors, such as specific cultural differences, in the effectiveness of injury-prevention and injury-reduction measures.
